# Legislation—Maryland

**Published:** 1896-01

**Authors:** A. W. Clement

**Affiliations:** Secretary


### LEGISLATION IN MARYLAND.

EDITOR JOURNAL OF COMPARATIVE MEDICINE.

DEAR SIR: The case of State of Maryland *vs.* J. C. Sigmund, practising as a veterinary surgeon without a license from the board, was decided in favor of the prosecution, thus establishing the constitutionality of the law. Dr. Sigmund had a diploma, but the diploma was issued by a school after January 1, 1895, which required but two years' course of study. The Maryland law requires three years' study and the passing of a satisfactory examination. Yours truly,

A. W. CLEMENT,
Secretary.

